# Supplementary material for: A Novel Method for Stimulating Cannabis sativa L. Male Flowers from Female Plants
Source: Plants (Basel). 2023 Sep 25;12(19):3371. doi: 10.3390/plants12193371 (PMC10574327; doi:10.3390/plants12193371)
Supplement: Supplementary file 1 [file plants-12-03371-s001.zip › plants-2570387-supplementary.pdf]

Table S1. Male flower counts of each treated plant

Plant accession	treatment	Flower counts
H2020-377-003	STS	585
H2020-377-003	STS	688
H2020-377-003	STS	405
H2021-001-006	STS	391
H2021-001-006	STS	420
H2021-001-006	STS	250
H2020-376-003	STS	595
H2020-376-003	STS	705
H2020-376-003	STS	500
H2020-424-001	STS	540
H2020-424-001	STS	485
H2020-424-001	STS	200
H2020-376-002	STS	565
H2020-376-002	STS	Dead
H2020-376-002	STS	275
H2020-425-015	STS	525
H2020-425-015	STS	850
H2020-425-015	STS	150
H2020-377-003	ALESCO®	300
H2020-377-003	ALESCO®	400
H2020-377-003	ALESCO®	725
H2021-001-006	ALESCO®	230
H2021-001-006	ALESCO®	410
H2021-001-006	ALESCO®	295
H2020-376-003	ALESCO®	660
H2020-376-003	ALESCO®	550
H2020-376-003	ALESCO®	500
H2020-424-001	ALESCO®	600
H2020-424-001	ALESCO®	525
H2020-424-001	ALESCO®	Dead
H2020-376-002	ALESCO®	400
H2020-376-002	ALESCO®	275
H2020-376-002	ALESCO®	625
H2020-425-015	ALESCO®	625
H2020-425-015	ALESCO®	1000
H2020-425-015	ALESCO®	350

Table S2. Pollen quality examinations by Acetocarmine and FDA stains.

Stain	Hemp ID	Treatment	Total pollen #	Aborted pollen	Stained pollen	Proportion %*
Acetocarmine	H2020_376_003	ALESCO®	1379	126	1253	90.86[a]
		STS	1129	87	1042	92.29[a]
	H2020_425_015	ALESCO®	1201	183	1018	84.76[b]
		STS	1078	218	860	79.78[b]
FDA	H2020_376_003	ALESCO®	1348	75	1273	94.44[A]
		STS	1371	160	1211	88.33[B]
	H2020_425_015	ALESCO®	939	205	734	78.17[C]
		STS	697	197	500	71.74[D]

*Upper and lower cases indicate two independent multiple comparisons.
